# Complete mitochondrial genome of the river stingray *Potamotrygon orbignyi* (Myliobatiformes: Potamotrygonidae)

**DOI:** 10.1080/23802359.2019.1666683

**Published:** 2019-09-20

**Authors:** David Ory, Yves Cuenot, Régis Vigouroux, Raphaël Covain, Sébastien Brosse, Jérôme Murienne

**Affiliations:** a Laboratoire Evolution et Diversité Biologique (UMR5174), CNRS, Université Toulouse 3 Paul Sabatier, IRD, Toulouse, France;; b Laboratoire HYDRECO, Laboratoire Environnement de Petit Saut, Kourou, French Guiana;; c Muséum d’histoire naturelle, Genève, Switzerland

**Keywords:** Genome skimming, Illumina Hiseq, shotgun sequencing

## Abstract

The river stingray *Potamotrygon orbignyi* is a carnivorous bottom feeder that is widespread in the Amazonian region. We here assemble the 17,449 bp complete mitochondrial genome of the species, showing a typical gene arrangement as for related Potamotrygonidae. The analysis of the COI gene confirmed the identification of the specimen as *P. orbignyi*. A phylogenetic analysis of all Potamotrygonidae complete mitochondrial genomes highlights the close relationship between *P. orbignyi* and *P. motoro*.

Exclusively distributed in river systems of South America, the family Potamotrygonidae is composed of two subfamilies: Styracurinae and Potamotrygoninae (De Carvalho et al. 2016). Four genera are presently recognized in the Potamotrygoninae subfamily, *Potamotrygon* being the most diverse and containing 36 species (Da Silva and Loboda 2019). *P. orbignyi* is a typical carnivorous bottom feeder and presents a widespread distribution in the upper, mid, and lower Amazon Basin, the Orinoco drainage and in the coastal rivers of the Guianas (Da Silva and De Carvalho 2015).

A specimen of *P. orbignyi* was collected using hook and line in 2015, from the Approuague River in French Guiana (N 4.1850, W -52.3529; upstream of “Saut Athanase”). After morphological identification, muscle tissue (voucher ID: FL-15-244) was preserved in 96% ethanol and stored in the collection of the EDB laboratory. We performed a genome-skimming strategy (Murienne et al. 2016) on a 1/24th of a lane of an Illumina HiSeq 3000 flow cell. The circular 17,449 bp mitochondrial genome (GenBank accession no. MN178254) was assembled using NOVOplasty (Diercksxens et al. 2017) and annotated using MitoAnnotator (Iwasaki et al. 2013). The minimum sequencing depth was 329 X and the maximum 906 X. The mitochondrial genome shows the typical gene arrangement for vertebrates. All protein-coding genes started with an ATG codon except for the cytochrome c oxidase subunit I (COI) gene who started by GTG codon. Seven TAA and two TAG stop codons were identified. Four incomplete stop codons were found (ND2, ND3, ND4, and COII) adjacent to transfer RNAs encoded on the same strand.

In order to validate the morphological identification of the specimen, we used the Barcoding of Life Database Identification Engine on the 5’ region of the COI sequence using BOLD webserver (Ratnasingham and Hebert 2007). The sequence was identified as *P*. *orbignyi* therefore confirming the morphological identification. The four best hits were specimens of *P. orbignyi* with genetic similarity ranging from 99.84% to 100%. We performed a phylogenetic analysis of all available Potamotrigonidae complete mitochondrial genomes. A partial mtDNA of *Potamotrygon hystrix* (JN184071) without 12S rRNA, 16S rRNA and ND6 gene sequences was also included. As no sequence of Styracurinae was available, we used *Dasyatis bennetti* (NC_020352), Dasyatidae, as outgroup. A maximum-likelihood phylogenetic analysis (Figure. 1) was performed on all the 13 protein-coding genes and rRNA using RAxML-ng (Kozlov et al. 2019) and a GTR + G model applied for each gene. The tree shows the monophyly of the genus *Potramotrygon* and the close relationship between *P. orbignyi* and *P. motoro*. Comparison of the genes sequences highlights a strong similarity (mean 98.5% identity) among the two species. We, therefore, encourage future studies to publish additional complete mitochondrial genome for *P. motoro*. This should allow to determine if the close relationship between the complete mitochondrial sequences is due to introgression, a misidentification of the specimen of *P. motoro* used by Song et al. (2015), or if *P. orbignyi* and *P. motoro* is an invalid species distinction for local populations belonging to a same species.

**Figure 1.**
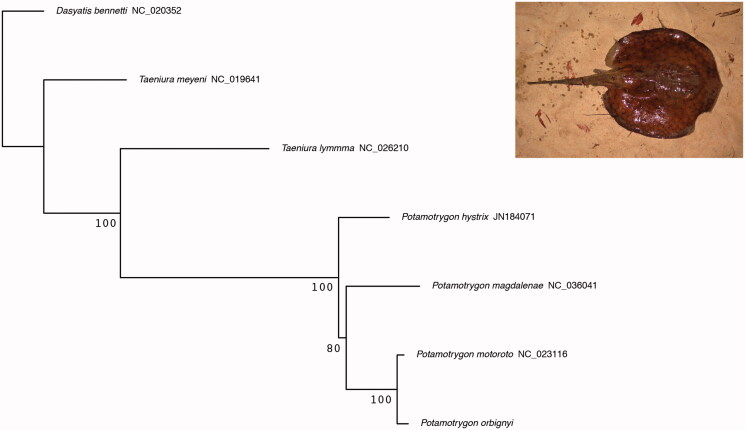
Maximum-likelihood phylogeny of the family Potamotrygonidae inferred from all available mitochondrial rRNAs and PCGs. Bootstrap support is indicated on nodes. Right panel: picture of the *P. orbignyi* specimen taken after capture.

## References

[CIT0001] Da Silva J, De Carvalho MR 2015 Systematics and morphology of *Potamotrygon orbignyi* (Castelnau, 1855) and allied forms (Chondrichthyes: Myliobatiformes: Potamotrygonidae). Zootaxa. 3982:1–082. 26250018 10.11646/zootaxa.3982.1.1

[CIT0002] Da Silva J, Loboda ST 2019 *Potamotrygon marquesi*, a new species of neotropical freshwater stingray (Potamotrygonidae) from the Brazilian Amazon Basin. J Fish Biol. 95:594–612. DOI: 10.1111/jfb.14050 31095730

[CIT0003] De Carvalho MR, Loboda TS, Da Silva J 2016 A new subfamily, Styracurinae, and new genus *Styracura*, for *Himantura schmardae* (Werner, 1904) and *Himantura pacifica* (Beebe & Tee-Van, 1941) (Condrichthyes: Myliobatiformes). Zootaxa. 4175:201–221. 27811760 10.11646/zootaxa.4175.3.1

[CIT0004] Dierckxsens N, Mardulyn P, Smits G 2017 NOVOPlasty: de novo assembly of organelle genomes from whole genome data. Nucleic Acids Res. 45:e18. 28204566 10.1093/nar/gkw955 PMC5389512

[CIT0005] Iwasaki W, Fukunaga T, Isagozawa R, Yamada K, Maeda Y, Satoh TP, Sado T, Mabuchi K, Takeshima H, Miya M, Nishida M 2013 MitoFish and MitoAnnotator: a mitochondrial genome database of fish with an accurate and automatic annotation pipeline. Mol Biol Evol. 30:2531–2540. 23955518 10.1093/molbev/mst141 PMC3808866

[CIT0006] Kozlov AM, Darriba D, Flouri T, Morel B, Stamatakis A 2019 RAxML-NG: a fast, scalable and user-friendly tool for maximum likelihood phylogenetic inference. Bioinformatics. :btz305 DOI: 10.1101/447110 PMC6821337 31070718

[CIT0007] Murienne J, Jeziorski C, Holota H, Coissac E, Blanchet S, Grenouillet G 2016 PCR-free shotgun sequencing of the stone loach mitochondrial genome (*Barbatula barbatula*). Mitochondrial DNA A. 27:4211. 10.3109/19401736.2015.1022744 26000945

[CIT0008] Ratnasingham S, Hebert P 2007 BOLD: the barcode of life data system (http://www.barcodinglife.org). Mol Ecol Notes. 7:355–364. 18784790 10.1111/j.1471-8286.2007.01678.x PMC1890991

[CIT0009] Song HM, Mu XD, Wei MX, Wang XJ, Luo JR, Hu YC 2015 Complete mitochondrial genome of the ocellate river stingray (*Potamotrygon motoro*). Mitochondrial DNA. 26:857–858. 24409899 10.3109/19401736.2013.861429

